# Calcium: magnesium intake ratio and colorectal carcinogenesis, results from the prostate, lung, colorectal, and ovarian cancer screening trial

**DOI:** 10.1038/s41416-019-0579-2

**Published:** 2019-09-23

**Authors:** Jing Zhao, Ayush Giri, Xiangzhu Zhu, Martha J. Shrubsole, Yixing Jiang, Xingyi Guo, Reid Ness, Douglas L. Seidner, Edward Giovannucci, Todd L. Edwards, Qi Dai

**Affiliations:** 1 Division of Epidemiology, Vanderbilt Ingram Cancer Center, Department of Medicine, Vanderbilt University School of Medicine, Vanderbilt University Medical Center, Nashville, TN USA; 2 Division of Epidemiology, Department of Medicine, Vanderbilt University Medical Center, Nashville, TN USA; 3 Department of Medicine, Marlene and Stewart Greenebaum Cancer Center, University of Maryland, Baltimore, MD USA; 4 Department of Medicine, Vanderbilt Center for Human Nutrition, Vanderbilt University Medical Center, Nashville, TN USA; 5 Departments of Nutrition and Epidemiology, Harvard School of Public Health, Boston, MA USA

**Keywords:** Risk factors, Cancer prevention

## Abstract

**Background:**

We aimed to evaluate the associations between calcium and various stages of colorectal carcinogenesis and whether these associations are modified by the calcium to magnesium (Ca:Mg) ratio.

**Methods:**

We tested our hypotheses in the prostate lung, colorectal and ovarian cancer screening trial.

**Results:**

Calcium intake did not show a dose–response association with incident adenoma of any size/stage (*P*-_trend_ = 0.17), but followed an inverse trend when restricted to synchronous/advanced adenoma cases (*P*-_trend_ = 0.05). This inverse trend was mainly in participants with Ca:Mg ratios between 1.7 and 2.5 (*P*-_trend_ = 0.05). No significant associations were observed for metachronous adenoma. Calcium intake was inversely associated with CRC (*P*-_trend_ = 0.03); the association was primarily present for distal CRC (*P*-_trend_ = 0.01). The inverse association between calcium and distal CRC was further modified by the Ca:Mg ratio (*P*-_interaction_ < 0.01); significant dose–response associations were found only in participants with a Ca:Mg ratio between 1.7 and 2.5 (*P*-_trend_ = 0.04). No associations for calcium were found in the Ca:Mg ratio above 2.5 or below 1.7.

**Conclusion:**

Higher calcium intake may be related to reduced risks of incident advanced and/or synchronous adenoma and incident distal CRC among subjects with Ca:Mg intake ratios between 1.7 and 2.5.

## Background

Colorectal cancer (CRC) arises from the malignant transformation of adenomatous polyps also known as adenomas.^1^ Endoscopy screening followed by removal of adenomas is currently the most effective prevention strategy available to reduce morbidity and mortality associated with CRC.^2^ However, CRC still remains the fourth most common incident cancer and the second most common cause of cancer death in the US.^3,4^ Therefore, the need for investigation into additional preventive strategies is crucial.

Evidence from clinical trials and observational studies has not been consistent on the effect of calcium in the aetiology of CRC. Colorectal carcinogenesis is a continuum of disease progression from benign adenomas to CRC.^5^ It is possible that calcium plays different roles in preventing formation of primary adenomas, preventing recurrence of adenomas (metachronous), and/or inhibiting carcinogenesis at more advanced stages of cancer progression. Although limited, some epidemiologic studies found calcium intake may be related to colorectal adenoma risk.^6–10^ Early randomised trials showed calcium supplementation reduced risk for metachronous colorectal adenoma;^11,12^ however, a larger trial failed to find such an association,^13^ and found supplementation of calcium alone or calcium plus vitamin D increased risk of sessile serrated adenomas or polyps during the extended follow-up.^14^ A pooled-analysis of ten cohort studies reported a modest 14% reduction in CRC risk for high calcium consumption, when comparing extreme quintiles of total calcium consumption.^15^ However, the Women’s Health Initiative Calcium and Vitamin D (CaD) supplementation trial did not find evidence to support a protective effect of CaD supplementation on CRC risk during an average follow-up period of 7 years.^16^

A growing body of literature suggests that a balance between calcium and magnesium intakes (the Ca:Mg ratio) may modify the associations between intakes of calcium and magnesium and risk for various outcomes, including gastrointestinal neoplasia^6,17,18^ and cancer mortality.^19^ A Ca:Mg ratio range between 1.7 and 2.6 has been suggested to be optimal for calcium intake for these outcomes.^6,17–19^ We reported from a randomised trial conducted among individuals with Ca:Mg ratios over 2.6 that reducing Ca:Mg ratios to ~2.3 through magnesium supplementation optimised vitamin D status.^20,21^ A lack of consideration for the presence of such an effect modification in previous studies evaluating calcium and colorectal carcinogenesis-related outcomes may provide some explanation for inconsistent findings in the literature. Using the data from the prostate, lung, colorectal and ovarian (PLCO) cancer screening trial, we systematically evaluated the associations between calcium intake and a series of prospectively followed outcomes in the adenoma-to-colorectal cancer continuum, including incident adenoma, recurrent/metachronous adenoma and incident colorectal carcinoma; and to evaluate whether these associations are modified by the Ca:Mg ratio.

## Methods

### Study design overview

**Fig. 1:**
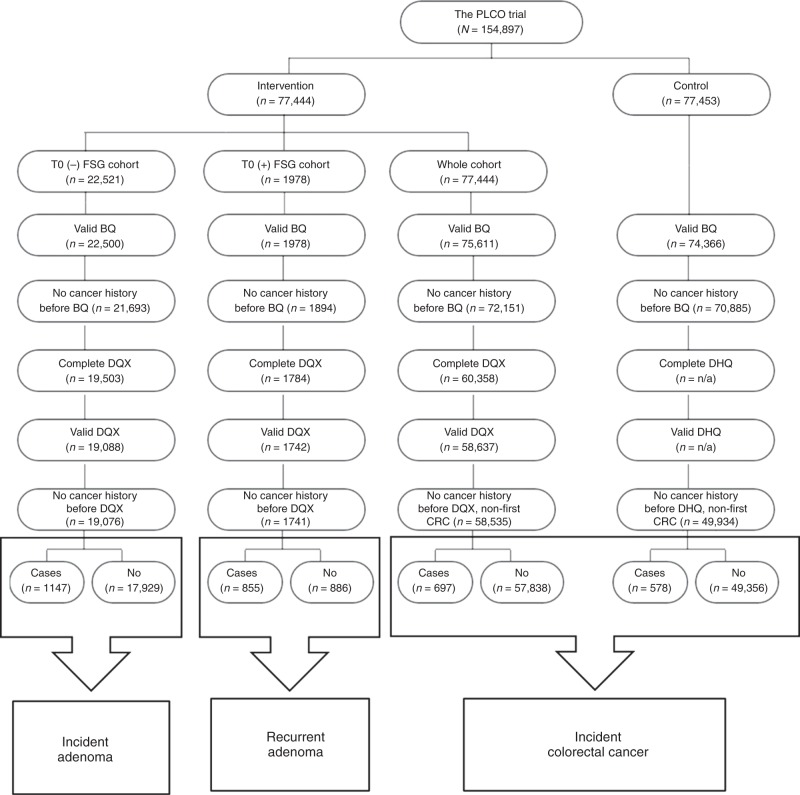
Flowchart detailing inclusion/exclusion criteria for case–control designs evaluating incident adenoma and recurrent adenoma and cohort design evaluating incident colorectal cancer

The PLCO is a multi-centre randomised, controlled trial comparing screening tests and usual care to determine the effects of screening on cancer-related mortalities associated with prostate, lung, colorectal and ovarian cancers. Details regarding PLCO study design were described extensively elsewhere.^22,23^ Briefly, around 155,000 eligible men and women aged 55–74 years were recruited and randomised between November 1993 and July 2001 across ten study centres. For the CRC endpoint, participants in the intervention received flexible 60-cm sigmoidoscopy (FSG) compared with usual care for participants in the control arm. Participants from both the intervention and control arms of CRC screening study were included in this sub-study. A detailed breakdown of study population and inclusion criteria for incident adenoma, recurrent/metachronous adenoma and incident colorectal carcinoma are described in Fig. 1.

At the entry of the trial, participants were asked to complete a Baseline Questionnaire (BQ). The BQ collected information on demography (e.g., age, sex, race, education and marital status), family cancer history, personal medical history (including histories of colorectal polyp, nonsteroidal anti-inflammatory drugs use and cancer screening history within 3 years), anthropometry (height and weight), lifestyle factors including smoking history. A total of 75,611 (97.6%) participants in the intervention arm and 74,366 (96.0%) participants in the control arm completed the BQ.

To capture average dietary intake spanning 12 months prior to enrolment, we used data collected in the Dietary Questionnaire (DQX) for the intervention arm and Dietary History Questionnaire (DHQ) for the control arm, both of which were administered at baseline. We also used the information collected at baseline on exercise levels from the DQX. DQXs or DHQs were considered invalid for any of the following four criteria^24^: (1) missing date of completion; (2) death before the completion of the DQX; (3) eight or more missing items and (4) extreme (highest or lowest 1% for each sex) caloric intake. Only valid dietary questionnaires were used for analyses which resulted in 58,535 (75.6%) in the intervention arm, and 49,934 (64.5%) in the control arm. The usual dietary intakes of nutrients including calcium were derived from frequency and portion-size responses from the food frequency questionnaire, in which nutrient values per portion were multiplied by the daily frequency of intake and summed across all relevant food items. Nutrient composition databases were constructed based on U.S. Department of Agriculture food composition tables (USDA’s 1994–1996 Continuing Survey of Food Intakes by Individuals [CSFII]). Supplement use was calculated from single and/or multi-vitamins use based on the responses to the supplement questions on the DQX or DHQ.

FSG was performed at study entry (T_0_) and then at the 3-(T_3_) or 5-(T_5_) year follow-up visits for participants in the intervention arm.^25,26^ In the intervention arm, ~65,000 subjects underwent baseline FSG, ~42,000 received it at visits T_3_ or T_5_ and ~39,000 participants who had FSGs both at baseline and at the T_3_ or T_5_ visits. An FSG result was defined as “positive” if one or more of the following were found: rectal nodule, rectal and/or colon mass and/or colon polyp. Participants with a positive FSG were referred to their health care providers for further diagnostic evaluation and follow-up. Study centres actively tracked subjects to collect, assemble, organise and abstract medical record information related to diagnosis during the follow-up. Within 12 months after a positive FSG, the PLCO study staff abstracted medical records pertaining to diagnostics.^25,26^

### Outcomes

#### Colorectal adenoma incidence

Participants (*n* = 22,521) with a negative T_0_ FSG in the intervention arm of the PLCO trial were eligible to be included in this sub-cohort for evaluation of incident colorectal adenoma risk. In addition, a T_3/5_ screen was required to further classify an incident adenoma case or control. Positive T_3/5_ screens which led to the discovery of a left-sided adenoma were considered cases.^26^ Controls needed to have a negative T_3/5_ FSG. After limiting analysis to individuals with a valid BQ (*n* = 22,500), no cancer history before BQ (*n* = 21,693), complete DQX (*n* = 19,503), a valid DQX (*n* = 19,088), and no cancer history before DQX (*n* = 19,076), 1147 incident colorectal adenoma cases and 17,929 control participants were identified. An adenoma with ≥ 1 cm in size, high-grade dysplasia or villous component was defined as an advanced adenoma.

#### Metachronous colorectal adenoma (recurrent adenoma)

Participants with a positive baseline FSG screening, diagnostic endoscopy within 6 months from baseline and no cancer findings were invited to complete the interviewer-administered telephone-based Study of Colonoscopy Utilization (SCU) questionnaire.^26^ A baseline adenoma was defined as an adenoma found within the first 18 months following a positive T_0_ FSG screen, on the first endoscopy that followed the T_0_ FSG screen, or on an endoscopy within 6 months of the first endoscopy following the screen. A questionnaire collected information on all known endoscopy after randomisation. Medical record abstraction was performed to verify the collected questionnaire information. Individuals with diagnosed adenoma at baseline but free of adenoma at the second endoscopy were considered controls for this analysis, while individuals with a diagnosis of adenoma at the second endoscopy after resection of adenoma found at baseline were defined as recurrent colorectal adenoma cases. Participants not in SCU but with a positive T_3/5_ screen which resulted in an endoscopy that discovered recurrence were also included.

After further restricting to individuals who completed a valid BQ (*n* = 1978), no cancer history before BQ (*n* = 1894), complete DQX (*n* = 1784), a valid DQX (*n* = 1742) and who had no cancer history before DQX (*n* = 1741), the final analysis included 855 colorectal metachronous adenoma cases and 886 controls.

#### CRC incidence

Over a median follow-up period of 12.5 years, colorectal cancer incidence was ascertained primarily through mailed Annual Study Update Questionnaire and repeated mailing or telephone for those who did not respond. Medical records were used to verify cancer incidence, stage and location.^25^ The intervention arm of the PLCO trial was further restricted to a valid BQ (*n* = 75,611), no history of any cancer prior to BQ (*n* = 72,151), completed a DQX (*n* = 60,358), has valid DQX (*n* = 58,637) and no history of any cancer prior to DQX (*n* = 58,535). The control arm of the PLCO trial was further restricted to a valid BQ (*n* = 74,366), no history of any cancer prior to BQ (*n* = 70,885) and no history of any cancer prior to DHQ (*n* = 49,934). The final analysis included 58,535 subjects in the intervention arm, of whom 697 developed CRC during follow-up. The control arm was reduced to 49,934 participants with valid BQ, DHQ and no cancer history, of whom 578 developed CRC during follow-up.

#### Statistical analysis

Summary statistics for both continuous (mean ± standard deviation) and categorical variables (count and percent) were used to describe study populations. Person-years for CRC incidence was calculated from the date of randomisation to the date of CRC diagnosis, death, loss-to-follow-up, or end of follow-up, whichever came first.^25^

Since information on incident and metachronous adenoma was only collected and confirmed after the T_3_ or T_5_ screen, we estimated 5-year risks for incident and metachronous adenomas with odds ratios and corresponding 95% confidence intervals (95% CIs) calculated using multivariable adjusted unconditional logistic regression. Risk for incident CRC was estimated using hazard ratios and corresponding 95% CIs from multivariable adjusted cox-proportional hazard models. Potential confounding factors were selected based on biological plausibility, literature reports and/or ≥10% change in relative risks.^27^ Confounding factors evaluated included age, sex, race, education, recruitment site, family history of CRC, body mass index, smoking status, alcohol consumption, exercise and daily intakes of total energy, vitamin D and magnesium. Tests for trend across categories were performed in regression models by assigning the score *j* to the *j*th level of the variable selected.

For primary analysis, calcium intake was categorised as 600 mg/day, 600–1200 mg/day, 1200–1600 mg/day and ≥1600 mg/day. Previous studies showed a protective effect of calcium in risk reduction at daily intake levels of calcium from 600 to 1000 mg/day,^28^ with no further protection beyond this range.^15,29,30^ Almost all participants in our study are 50 years or older. The calcium RDA is 1200 mg/day for women between 51 and 70 years and for all adults aged > 70 years.^31^ Thus, 600–1200 mg/day is used as the reference group. The cut-off at 1600 mg/day is the upper quartile in this study. Investigation of associations between calcium intake and all three outcomes were also conducted by strata of Ca:Mg ratios (<1.7, 1.7–2.5 and ≥2.5). Multiplicative interactions between calcium and the Ca:Mg ratio in relation to the three outcomes were formally tested using the likelihood ratio test or Wald test, where both variables, calcium and the Ca:Mg ratio, were treated as continuous variables for maximal power. To better evaluate the robustness of observed associations, several sensitivity and sub-group analyses were performed. For incident adenoma, in addition to evaluating adenoma of any size, sub-analyses were performed to evaluate associations with advanced/synchronous adenomas. For metachronous adenoma and CRC incidence in the intervention arm, analyses were stratified on baseline adenoma characteristics (e.g., advanced and/or synchronous adenoma). For CRC, analyses were performed by location of cancer: distal vs. proximal, and by clinical trial assignment: intervention arm vs. control arm. Finally, associations between calcium intake and the three outcomes were modelled as joint categories of Ca intake and magnesium intake as defined by the Recommended Dietary Allowance (RDA) (below RDA; at or above RDA). RDA for magnesium is 320 and 420 mg for women and men, respectively while RDA for calcium aged > 50 is 1200 and 1000 mg for women and men, respectively. All tests were two-sided, and statistical significance threshold was set at 0.05. Statistical analyses were performed using SAS statistical software (version 9.4; SAS Institute, Cary, NC).

## Results

### Incident colorectal adenoma

Compared with controls without polyps, incident adenoma cases were more likely to be male, smokers, physically inactive, have higher body mass index, and have higher intake of energy, but had lower intakes of calcium, magnesium and vitamin D (Table 1). No significant dose–response inverse association was observed between calcium intake and risk of incident colorectal adenoma (Table 2). However, intakes of calcium between 1200 mg and 1600 mg per day were associated with a significantly reduced risk of incident adenoma with an OR of 0.82 (95% confidence intervals (CI): 0.68–0.97) when compared with calcium intake between 600 and 1200 mg per day (referent group). When analyses were limited to incident advanced and/or synchronous adenomas, the inverse trend of associations was found with a corresponding OR of 0.71 (95% CIs: 0.52–0.96) for calcium intake between 1200 and 1600 mg. When these analyses were stratified by the Ca:Mg ratio, the dose–response between calcium intake and advanced and/or synchronous adenoma were only observed in participants with a Ca:Mg ratio between 1.7 and 2.5 (*P*-_trend_, 0.05). The number of cases in the Ca:Mg ratio < 1.7 strata was too small to make meaningful statistical inference. There was no statistically significant interaction between calcium and the Ca:Mg ratio (*P*-_interaction_: 0.11).

**Table 1:** Selected descriptive characteristics of by outcome status for incident adenoma, metachronous adenoma and incident colorectal cancer

Characteristics	Adenoma^a^		Metachronous adenoma^a^		Colorectal cancer^b^	
	Cases (*N* = 1147)	Controls (*N* = 17,929)	Cases (*N* = 855)	Controls (*N* = 886)	Cases (*N* = 1275)	Cohort^b^ (*N* = 107,194)
Age (years), mean ± SD	62.1 ± 5.3	62.3 ± 5.2	62.9 ± 4.8	62.7 ± 5.2	64.2 ± 5.3	62.6 ± 5.3
Sex, %						
Men	67.5	55.9	72.4	60.0	56.8	49.6
Race, %						
White	90.6	88.9	93.0	96.1	89.7	90.9
Education, %						
College or higher	38.7	39.5	38.0	35.7	31.7	36.1
Smoking status, %						
Never smoker	42.6	53.4	34.5	37.5	44.4	47.2
Former smoker	47.3	41.4	52.1	49.3	45.3	43.2
Current smoker	10.1	5.2	13.4	13.2	10.3	9.6
Alcohol consumption, %	81.1	79.1	84.0	83.1	75.9	75.3
Family history of colorectal cancer, %	10.3	8.9	13.2	13.7	13.4	10.5
Aspirin use, %	48.4	46.8	44.7	43.9	44.0	47.2
Physically inactive^c^, %	16.6	12.0	15.1	15.2	–	–
Body mass index ≥ 30, %	25.0	21.7	27.7	23.4	26.0	23.6
Daily nutrients intake, mean ± SE						
Total energy (kcal)^d^	2187 ± 24	2087 ± 6	2186 ± 28	2089 ± 27	1936 ± 22	1902 ± 2
Total calcium (mg)^e^	1161 ± 16	1255 ± 4	1119 ± 17	1196 ± 16	1057 ± 14	1130 ± 2
Total magnesium (mg)^e^	431.6 ± 3	444 ± 1	431 ± 4	439 ± 4	395 ± 3	404 ± 0.3
Vitamin D (mcg/day)^e^	10.8 ± 0.3	12.1 ± 0.1	10.4 ± 0.3	11.0 ± 0.3	10.2 ± 0.2	10.8 ± 0.02

^a^Adenoma and metachronous adenoma analyses were set up in a case–control framework and assessed with logistic regression

^b^Colorectal cancer analyses was set up as a cohort study with cases representing incident colorectal cancer and the cohort representing the remaining individuals who did not develop colorectal cancer during follow-up

^c^The information on physical activity is not available in the control arm

^d^Least squares mean value, SE

^e^Least squares mean value, SE, adjusting for total energy

**Table 2:** Association^a^ between calcium intake and colorectal adenoma incidence by calcium to magnesium intake ratio categories

Ca Intake (mg/day)	Any incident adenoma		Advanced and/or synchronous incident adenoma	
	Cases	OR (95% CI)	Cases	OR (95% CI)
All				
<600	139	0.89 (0.72–1.12)	55	1.17 (0.82–1.68)
600–1200	545	1.00 (Ref.)	192	1.00 (Ref.)
1200–1600	219	0.82 (0.68–0.97)	70	0.71 (0.52–0.96)
≥ 1600	244	0.84 (0.69–1.03)	83	0.80 (0.58–1.11)
* P-*_trend_^b^		0.17		0.05
Ca:Mg ratio is <1.7				
< 600	62	0.87 (0.55–1.38)	22	0.57 (0.29–1.14)
600–1200	71	1.00 (Ref.)	36	1.00 (Ref.)
1200–1600	3	0.57 (0.16–2.00)	1	0.37 (0.05–2.99)
≥ 1600	2	4.67 (0.80–27.35)	0	–
* P-*_trend_^b^		0.55		0.32
Ca:Mg ratio is between 1.7 and 2.5				
< 600	64	0.91 (0.66–1.26)	31	1.50 (0.92–2.47)
600–1200	284	1.00 (Ref.)	93	1.00 (Ref.)
1200–1600	62	0.72 (0.51–1.02)	19	0.60 (0.33–1.08)
≥ 1600	35	0.91 (0.55–1.51)	11	0.64 (0.27–1.54)
* P-*_trend_^b^		0.61		0.05
Ca:Mg ratio is >2.5				
<600	13	0.86 (0.47–1.60)	2	0.45 (0.11–1.88)
600–1200	190	1.00 (Ref.)	63	1.00 (Ref.)
1200–1600	154	0.81 (0.64–1.03)	50	0.74 (0.50–1.12)
≥1600	207	0.81 (0.62–1.06)	72	0.77 (0.49–1.22)
* P-*_trend_^b^		0.16		0.43
* P-*_interaction_^c^		0.94		0.11

^a^Adjusted for age (continuous), sex, BMI (<25, 25–30, ≥30), education (less than high school, 12 years or completed high school, post high school training other than college, some college, college graduate, postgraduate), race (white, black, Asian or others), family history of colorectal cancer (yes or no), cigarette (never smoked cigarettes, current or former), hours spent in vigorous activities (<1 h/week, 1 h/week, 2 h/week, 3 h/week, 4 + h/week) and total energy and vitamin D intake

^b^Assigned the score *j* to the *j*^th^ level of calcium intake and evaluated the significance of Wald test

^c^Estimated the full model with interaction term of calcium intake and Ca:Mg ratio and without this term in reduce model using likelihood ratio test

### Metachronous (recurrent) adenoma

Compared with participants without metachronous adenoma, metachronous adenoma cases were also more likely to be male and obese, and to have higher total energy intake but lower calcium intake (Table 1). Metachronous adenoma cases were less likely to be white and to have family history of CRC.

We did not observe any statistically significant associations between calcium intake and metachronous adenoma (Table 3). Associations were no different when analyses were broken down by strata of Ca:Mg intake ratio (Table 3), advanced adenoma or synchronous adenoma (Table 3), location of adenoma (i.e., distal or proximal, data not shown) and baseline adenoma characteristics (i.e., advanced/synchronous adenoma) (Supplementary Table 1).

**Table 3:** Association^a^ between calcium intake and colorectal metachronous adenoma incidence by calcium to magnesium intake ratio categories

Calcium Intake (mg/day)	Any metachronous adenoma		Advanced and/or metachronous adenoma	
	Cases	OR (95% CI)	Cases	OR (95% CI)
All				
<600	124	1.23 (0.87–1.73)	65	1.45 (0.96–2.19)
600–1200	393	1.00 (Ref.)	198	1.00 (Ref.)
1200–1600	190	1.02 (0.77–1.34)	96	0.97 (0.69–1.38)
≥1600	148	0.83 (0.60–1.15)	81	0.92 (0.61–1.37)
* P-*_trend_^b^		0.15		0.21
Ca:Mg ratio is <1.7				
<600	52	1.38 (0.63–3.02)	29	1.22 (0.47–3.22)
600–1200	62	1.00 (Ref.)	33	1.00 (Ref.)
1200–1600	4	0.45 (0.09–2.15)	2	0.74 (0.09–5.91)
≥1600	2	0.45 (0.09–2.15)	2	–
* P-*_trend_^b^		0.99		0.45
Ca:Mg ratio is between 1.7 and 2.5				
<600	63	1.33 (0.82–2.17)	32	1.44 (0.80–2.62)
600–1200	194	1.00 (Ref.)	91	1.00 (Ref.)
1200–1600	53	0.90 (0.52–1.56)	24	1.03 (0.51–2.07)
≥1600	22	2.34 (0.82–6.68)	11	3.32 (0.95–11.64)
* P-*_trend_^b^		0.89		0.90
Ca:Mg ratio is >2.5				
<600	9	0.85 (0.32–2.27)	4	0.76 (0.22–2.59)
600–1200	137	1.00 (Ref.)	74	1.00 (Ref.)
1200–1600	133	1.02 (0.69–1.50)	70	0.86 (0.53–1.38)
≥1600	124	0.73 (0.47–1.13)	68	0.65 (0.37–1.13)
* P-*_trend_^b^		0.21		0.18
* P-*_interaction_^c^		0.52		0.41

^a^Adjusted for age (continuous), sex, BMI (<25, 25–30, ≥30), education (less than high school, 12 years or completed high school, post high school training other than college, some college, college graduate, postgraduate), race (white, black, asian or others), family history of colorectal cancer (yes or no), cigarette (never smoked cigarettes, current or former), hours spent in vigorous activities (<1 h/week, 1 h/week, 2 h/week, 3 h/week, 4 + h/week) and total energy, magnesium and vitamin D intake

^b^Assigned the score *j* to the *j*^th^ level of calcium intake and evaluated the significance of Wald test

^c^Estimated the full model with interaction term of calcium intake and Ca:Mg ratio and without this term in reduce model using likelihood ratio test

### CRC incidence

Compared with participants who did not develop CRC during follow-up, incident CRC cases at baseline were more likely to be older, male, less likely to have attended college, less likely to be aspirin users, more likely to have history of CRC, and have higher body mass index and had lower intakes of calcium, magnesium and vitamin D (Table 1). Distribution of participant characteristics by calcium intake categories are detailed in Supplementary Table 2.

We found calcium intake was associated with a reduced risk of CRC (*P*-_trend_, 0.03) (Table 4). Closer examination of this association showed that the inverse trend between higher calcium categories and CRC was primarily present for distal CRC (*P*-_trend_, <0.01), but not for proximal CRC. In analysis stratified by the Ca:Mg intake ratio, we found the inverse trend between calcium intake and distal CRC was most pronounced in participants whose Ca:Mg ratio ranged from 1.7 to 2.5 (*P*-_trend_, 0.04). There was a statistically significant interaction between continuously modelled calcium intake and continuously modelled Ca:Mg ratio in relation to distal CRC (*P*-_interaction_, <0.01). When we further evaluated the relationship between calcium intake and distal CRC by randomisation status, the inverse trend across categories of calcium were similar in both groups, however, the association was statistically significant in the control arm (*P*-_trend_ < 0.01), but not in the intervention arm (*P*-_trend_ = 0.06) (Supplementary Table 3). Finally, when analyses were stratified by features of the baseline adenomas among individuals in the intervention arm, higher calcium intake trended towards reduced risk of CRC in individuals who had advanced/synchronous adenoma at baseline (*P*-_trend_ = 0.04) (Supplementary Table 1). Comparable data were not available in the control arm.

**Table 4:** Association^a^ between calcium intake and colorectal cancer incidence by calcium to magnesium intake ratio categories

Calcium Intake (mg/day)	Colorectal cancer incidence		Proximal colon cancer		Distal colon cancer	
	Cases	HR (95% CI)	Cases	HR (95% CI)	Cases	HR (95% CI)
All						
<600	274	1.12 (0.95–1.33)	133	0.94 (0.74–1.19)	141	1.38 (1.08–1.76)
600–1200	557	1.00 (Ref.)	302	1.00 (Ref.)	254	1.00 (Ref.)
1200–1600	227	0.85 (0.72–1.01)	134	0.94 (0.75–1.17)	93	0.75 (0.58–0.97)
≥1600	217	0.89 (0.73–1.07)	121	0.97 (0.75–1.26)	92	0.75 (0.56–1.01)
* P-*_trend_^b^		0.03		0.99		<0.01
Ca:Mg ratio is <1.7						
<600	148	1.28 (0.87–1.88)	73	0.99 (0.57–1.75)	75	1.53 (0.90–2.62)
600–1200	75	1.00 (Ref.)	74	1.00 (Ref.)	41	1.00 (Ref.)
1200–1600	2	0.44 (0.10–1.86)	1	0.75 (0.09–5.90)	1	0.30 (0.04–2.27)
≥1600	1	1.87 (0.24–14.57)	1	8.57 (0.98–74.79)	0	–
* P-*_trend_^b^		0.18		0.78		0.06
Ca:Mg ratio is between 1.7 and 2.5						
<600	101	1.12 (0.85–1.47)	50	0.95 (0.65–1.39)	51	1.34 (0.90–1.99)
600–1200	269	1.00 (Ref.)	144	1.00 (Ref.)	124	1.00 (Ref.)
1200–1600	51	0.70 (0.49–1.01)	28	0.80 (0.48–1.33)	23	0.62 (0.37–1.06)
≥1600	21	0.89 (0.51–1.55)	11	1.19 (0.56–2.54)	9	0.59 (0.25–1.39)
* P-*_trend_^b^		0.17		0.94		0.04
Ca:Mg ratio is >2.5						
<600	25	1.16 (0.75–1.79)	10	0.78 (0.41–1.51)	15	1.76 (0.98–3.14)
600–1200	213	1.00 (Ref.)	124	1.00 (Ref.)	89	1.00 (Ref.)
1200–1600	174	0.91 (0.73–1.13)	105	0.97 (0.73–1.29)	69	0.83 (0.59–1.17)
≥1600	195	0.93 (0.73–1.20)	109	0.97 (0.70–1.35)	83	0.84 (0.57–1.24)
* P-*_trend_^b^		0.41		0.93		0.12
* P-*_interaction_^c^		0.08		0.64		<0.01

^a^Adjusted for arm, age (continuous), sex, BMI (<25, 25–30, ≥30), education (less than high school, 12 years or completed high school, post high school training other than college, some college, college graduate, postgraduate), race (white, black, asian or others), family history of colorectal cancer (yes or no), cigarette (never smoked cigarettes, current or former), hours spent in vigorous activities (<1 h/week, 1 h/week, 2 h/week, 3 h/week, 4 + hours/week), and total energy and vitamin D intake

^b^Assigned the score *j* to the *j*^th^ level of calcium intake and evaluated the significance of Wald test

^c^Estimated the full model with interaction term of calcium intake and Ca:Mg ratio and without this term in reduce model using likelihood ratio test

## Discussion

Considering the inconsistent evidence in the literature regarding the association between calcium intake and colorectal carcinogenesis, we designed this study to address two broad questions, that, if calcium intake is protective against colorectal carcinogenesis, at which stage(s) in the carcinogenesis process is this association most evident, and is the association of this presumed protection modified by a balance between the intake ratios of Ca and Mg. Although intake of calcium did not have a dose–response relationship with incident adenoma of any size, we observed an inverse trend when considering only incident advanced and/or synchronous adenomas. We did not find evidence of association between calcium intake and metachronous adenoma. We noted an inverse trend between calcium intake and CRC and this trend was notably driven by associations with distal CRC rather than with proximal CRC. We then tested if the inverse associations noted above were dependent on an optimally balanced Ca:Mg intake ratio. Interestingly, the inverse trends noted for calcium intake with regards to incident advanced adenoma and incident distal CRC were significant when the Ca:Mg intake ratio was between 1.7 and 2.5.

The results from our study are in agreement with some previously published studies, but in conflict with others. Both cohort studies and earlier intervention trials found high calcium intake or supplementation was related to moderately reduced risks of adenoma, metachronous adenoma and CRC.^7–12,15,32^ However, the results from a large-scale randomised clinical trial (WHI) do not support an effect of calcium plus vitamin D supplementation on the incidence of CRC after 7 years of follow-up.^16^ It is possible that the follow-up period in WHI may not have been long enough for a protective effect to be evident. In fact, the investigation of the Nurses’ Health Study and Health Professionals Follow-up Study not only showed an inverse association between calcium intake and CRC but also showed that the associations became progressively stronger with increasing periods of latency, and was strongest for the 12–16 years follow-up period.^33^ With an average follow-up period more similar to this study than the WHI, we too report similar point estimates as Zhang et al., with a stronger inverse association for distal CRC but not for proximal CRC.^33^ A pooled-analysis of ten cohort studies also reported the inverse association between milk and CRC was limited to cancers of the distal, not the proximal colon.^15^ Thus accumulating evidence consistently indicates calcium intake is associated with reduced risk of distal CRC.

CRC is believed to arise, in the overwhelming majority of cases, from adenomas via the well-established adenoma-carcinoma sequence.^34^ Findings from this study may provide possible explanations for inconsistent results in previous studies evaluating the effect of calcium on colorectal carcinogenesis.^6,8–10,35,36^ An earlier study hypothesised that the chemo-preventive effects of calcium intake on CRC may primarily exert its effects only in early stages (i.e., adenoma).^16^ Our findings are consistent with previous epidemiologic data,^15,37^ suggesting higher calcium intake may only inhibit early colorectal carcinogenesis at the stage of incident adenoma^6–10,15^ and the association may be stronger for prevention of incident advanced adenoma, a premalignant lesion for CRC,^15^ than other types of adenoma/polyps.^38^ The possibility is consistent with the observation that the magnitude of reduction in overall CRC risk associated with high calcium intake is similar to the reduction in adenoma risk.

In this study, we did not observe any meaningful associations or trends between calcium intake and metachronous adenomas. However, of the three outcomes we evaluated, sample size and statistical power were also the smallest for this analysis. Although earlier randomised trials found calcium supplementation reduced risk of colorectal metachronous adenoma,^11^ a recent trial of calcium supplementation failed to find such an association.^13^ In fact, the trial found supplementation of calcium alone or calcium plus vitamin D substantially increased risk of sessile serrated adenomas or polyps during the extended follow-up.^14^ Other underlying factors may account for the inconsistency between these randomised trials, such as separating sessile serrated adenomas or polyps from adenoma or polyps and the change in the Ca:Mg intake ratios over the time. The Ca:Mg intake ratio in the study populations has increased from ~2.6 in earlier trials to >3.0 in recent years.^11,12,39^ A key goal of this study was to investigate whether an optimal Ca:Mg ratio enhances the protective associations between calcium and colorectal outcomes. Working within the constraints of the data set while incorporating knowledge from prior studies, we set the Ca:Mg ratio cut-points at 1.7, the lower bound of the Ca:Mg ratio, below which calcium intake has not found to be beneficial,^18^ and 2.5, the median, which also approximates the upper bound of the beneficial Ca:Mg ratio proposed in prior studies at 2.6.^17^

It is possible that 2.5 may not serve as the optimal Ca:Mg ratio cut point to differentiate adequate vs. inadequate Ca:Mg ratios. It is also notable that the magnitudes of the inverse associations between calcium and distal CRC are weaker in the >2.5 Ca:Mg ratio category than compared with the middle category (1.7–2.5). The Ca:Mg ratio strata of <1.7 had too few observations to make explicit extrapolations. Nonetheless, the waning of the observed inverse association between calcium and distal CRC with increasing Ca:Mg ratio categories is also reflected in the positive beta estimate for the interaction term when calcium and Ca:Mg ratio were modelled as continuous variables (data not shown). Thus, our results suggest that the optimal Ca:Mg ratio may be located somewhere between 1.7 and 2.5.

In an earlier study, we reported that the dietary intake ratio of Ca:Mg modified the association between calcium, magnesium and prevalent colorectal adenoma.^6^ In a subsequent randomised clinical trial, calcium supplementation only reduced risk of metachronous colorectal adenoma when the baseline Ca:Mg ratio was <2.63.^17^ We found that the Ca:Mg ratio modified the associations between intakes of calcium and magnesium and risk of oesophageal neoplasia.^18^ A case–control study conducted in Belgium reported that a high calcium intake with a low magnesium intake was associated with increased risk of bladder cancer.^40^ In studies conducted in East Asian populations with a low Ca:Mg intake ratio (a median around 1.7), the association between intakes of calcium and magnesium and several outcomes (total, cardiovascular and/or cancer mortalities) were modified by the Ca:Mg ratio, but not by calcium or magnesium intake alone.^19^ In a randomised trial, we found reducing Ca:Mg ratios to around 2.3 through magnesium supplementation optimised vitamin D status (i.e., increasing blood 25-hydroxyvitamin D3 (25(OH)D3) when baseline 25(OH)D levels were lower, but decreasing 25(OH)D3 when baseline 25(OH)D were higher).^20,21^ Thus, the optimal balance between calcium and magnesium intake is a critical factor to consider in the investigation of associations between intakes of calcium and magnesium and cancer development.

It is noteworthy that when we evaluated the joint associations of Ca intake with Mg intake categorised by RDA level for three different types of colorectal neoplasia risk (incident adenoma, incident CRC or incident distal CRC), we found similar trends as in our primary analyses where the inverse associations for Ca intake in relation to these outcomes were all stronger for Mg intake at or above the RDA compared to Mg intake less than the RDA (Supplementary Tables 4–6). However, none of the tests for interaction were statistically significant in contrast to the tests for interaction we observed with the Ca:Mg ratios. This is consistent with previous findings in East Asian populations in which the interactions were statistically significant between Ca:Mg ratios with calcium intake, but not between magnesium intake with calcium intake.^19^ In other words, the interaction with Ca:Mg ratios cannot be explained by magnesium intake alone.

To our knowledge, no previous study has prospectively evaluated the associations of calcium intake with incident adenoma, metachronous adenoma, and incident cancer in the same cohort using the same food frequency questionnaire, which minimises misclassification that can occur when combining dietary estimates from multiple studies with different food frequency questionnaire instruments. The PLCO screening trial provides a unique and unparalleled opportunity to prospectively and concurrently evaluate these associations. One weakness is that the PLCO is a sigmoidoscopy-based randomised trial, which only screened the distal colorectal region. Thus, incident adenoma cases mainly included adenomas from the left side of the bowel although metachronous adenoma and incident CRC cases included cases from both proximal and distal regions. However, it is interesting that reports found that colonoscopy was linked to reduced deaths from the distal colorectal region, but not the right.^41,42^ Similarly another report from the PLCO observed sigmoidoscopic screenings only reduced distal, but not proximal CRC mortality.^25^ Further, sigmoidoscopic screenings also significantly reduced both distal and proximal CRC incidence in the PLCO.^25^ Thus, the PLCO provides a unique opportunity to test whether calcium intake confers additional protection against proximal and distal CRC among those receiving sigmoidoscopic screening(s). Similar to other nutritional epidemiological studies using food frequency questionnaires, there are possibly non-differential measurement errors which usually bias the results to the null. In addition, despite the relatively large sample sizes in our study, our investigations between calcium intake and colorectal carcinogenesis were constrained when evaluating by Ca:Mg ratio strata. The number of cases for some sub-categories of calcium intake in the <1.7 Ca:Mg ratio stratum were few and in some instances zero, making inferences relating to trends difficult in this stratum. Furthermore, to maintain relatively balanced number of cases in calcium categories in the largest Ca:Mg ratio stratum, we choose 2.5, the median in the data set, as one of the Ca:Mg ratio cut-offs, instead of 2.6 as investigated in previous studies. Finally, considering investigation of three main outcomes, several sub-group analyses and tests for effect modification, it is possible that findings reported in this study may be due to chance. We found different calcium intake distribution between intervention arm and control arm shown in Supplementary Table 1. The reason is not clear, and it may be due to different food frequency questionnaires were in two arms. However, we found the association pattern between calcium intake and CRC risk is similar in the intervention arm and control arm.

## Conclusion

In conclusion, findings from our study suggest that total calcium intake may reduce risk for CRC, particularly in the distal colorectal region and that this reduced risk may be conferred during the earlier stage of carcinogenesis, for any or advanced adenomas. Finally, our study adds to the growing evidence which suggests a balance between calcium and magnesium intake, specifically calcium intake at dietary reference intake levels while maintaining a Ca:Mg intake ratio between 1.7 and 2.5, may be important for CRC prevention, and warrants further research in a large randomised trial setting.

## Supplementary information

Supplementary Tables

## Data Availability

Researchers can submit applications to the National Cancer Institute to use the PLCO data.
