# Supplementary material for: Calcium: magnesium intake ratio and colorectal carcinogenesis, results from the prostate, lung, colorectal, and ovarian cancer screening trial
Source: Br J Cancer. 2019 Sep 23;121(9):796–804. doi: 10.1038/s41416-019-0579-2 (PMC6889387; doi:10.1038/s41416-019-0579-2)
Supplement: Supplementary file 1 — Supplementary Tables [file 41416_2019_579_MOESM1_ESM.docx]

**Supplementary Table 1. Sensitivity analyses: Association between calcium intake and metachronous adenoma or colorectal cancer in the intervention arm by baseline advanced/synchronous adenoma status (absence/presence)**

| Ca Intake (mg/day) | Metachronous adenoma | |  | Colorectal Cancer in intervention arm | |
| --- | --- | --- | --- | --- | --- |
|  | Cases | OR (95% CI) |  | Cases | HR (95% CI) |
| **No advanced/synchronous adenoma at baseline** | | | | | |
| <600 | 37 | 1.92 (1.02-3.59) |  | 73 | 0.98 (0.74-1.30) |
| 600 -1200 | 98 | 1.00 (Ref) |  | 245 | 1.00 (Ref) |
| 1200 -1600 | 46 | 0.97 (0.57-1.66) |  | 107 | 0.86 (0.67-1.10) |
| ≥1600 | 41 | 0.97 (0.52-1.81) |  | 133 | 0.99 (0.76-1.28) |
| *P* _trend_^2^ |  | 0.26 |  |  | 0.80 |
| **Has advanced/synchronous adenoma at baseline** | | | | | |
| <600 | 87 | 1.03 (0.70-1.52) |  | 21 | 1.46 (0.83-2.59) |
| 600 -1200 | 295 | 1.00 (Ref) |  | 51 | 1.00 (Ref) |
| 1200 -1600 | 144 | 1.01 (0.73-1.40) |  | 14 | 0.52 (0.26-1.00) |
| ≥1600 | 107 | 0.78 (0.54-1.14) |  | 17 | 0.66 (0.34-1.30) |
| *P* _trend_^2^ |  | 0.28 |  |  | 0.04 |
| *P* _interaction_^3^ |  | 0.54 |  |  | 0.09 |

^1^ Adjusted for age (continuous), sex, BMI (<25, 25-30, ≥30), education (less than high school, 12 years or completed high school, post high school training other than college, some college, college graduate, postgraduate), race (white, black, asian or others), family history of colorectal cancer (yes or no), cigarette (never smoked cigarettes, current or former), hours spent in vigorous activities (less than1 hour/week, 1 hour/week, 2 hours/week, 3 hours/week, 4+ hours/week), and total energy and vitamin D intake

^2^ Assigned the score j to the j^th^ level of calcium intake and evaluated the significance of Wald test

^3^ Estimated the full model with interaction term of calcium intake and Ca:Mg ratio and without this term in reduced model using likelihood ratio test

| **Supplementary Table 2. Selected descriptive characteristics of eligible cohort participants by calcium intake (mg/day)** | | | | | | | | | | | |
| --- | --- | --- | --- | --- | --- | --- | --- | --- | --- | --- | --- |
| Characteristics | Intervention arm | | | | |  | Control arm | | | | |
|  | < 600 | 600-1200 | *1200-1600* | *≥1600* | *P* |  | < 600 | 600-1200 | *1200-1600* | *≥1600* | *P* |
| N | 7,323 | 24,475 | 12,354 | 14,383 |  |  | 12,678 | 21,185 | 9,584 | 6,487 |  |
| No. of colorectal cancer cases | 100 | 315 | 128 | 154 |  |  | 174 | 242 | 99 | 63 |  |
| Person-years | 80,222 | 273,937 | 138,958 | 161,286 |  |  | 142,777 | 241,744 | 110,113 | 74,392 |  |
| Age (years) |  |  |  |  |  |  |  |  |  |  |  |
| Mean^1^ | 62.6 | 62.7 | 62.6 | 62.6 |  |  | 62.6 | 62.5 | 62.5 | 62.5 |  |
| SE | 0.06 | 0.03 | 0.05 | 0.04 | 0.12 |  | 0.05 | 0.04 | 0.05 | 0.07 | <.0001 |
| Sex (men), % | 53.7 | 58.6 | 50.2 | 39.2 | <.0001 |  | 55.9 | 52.2 | 36.1 | 34.0 | <.0001 |
| Race (white), % | 81.3 | 90.2 | 93.1 | 94.7 | <.0001 |  | 85.2 | 91.9 | 93.4 | 95.7 | <.0001 |
| Education, % |  |  |  |  |  |  |  |  |  |  |  |
| College or higher | 48.5 | 56.9 | 59.3 | 61.1 | <.0001 |  | 54.0 | 57.9 | 59.3 | 61.1 | <.0001 |
| Smoking status, % |  |  |  |  |  |  |  |  |  |  |  |
| Never smoker | 42.4 | 44.5 | 47.9 | 52.2 |  |  | 44.0 | 47.1 | 50.6 | 51.6 |  |
| Former smoker | 44.0 | 44.8 | 43.3 | 40.5 |  |  | 44.4 | 43.9 | 41.3 | 40.9 |  |
| Current smoker | 13.6 | 10.7 | 8.8 | 7.3 | <.0001 |  | 11.6 | 9.0 | 8.1 | 7.5 | <.0001 |
| Alcohol consumption, % | 75.6 | 78.7 | 77.8 | 76.2 | <.0001 |  | 72.8 | 73.8 | 72.5 | 69.7 | <.0001 |
| Family history of colorectal cancer, % | 10.3 | 10.9 | 10.5 | 11.0 | 0.0038 |  | 9.9 | 10.3 | 10.2 | 10.4 | 0.61 |
| Aspirin use, % | 43.0 | 46.4 | 49.1 | 49.3 | <.0001 |  | 45.2 | 47.7 | 47.5 | 48.5 | <.0001 |
| Body mass index ≥ 30, % | 25.1 | 24.1 | 24.3 | 22.7 | <.0001 |  | 24.5 | 23.8 | 21.2 | 22.3 | <.0001 |
| Total energy (kcal)^2^ |  |  |  |  |  |  |  |  |  |  |  |
| Mean^1^ | 1,341 | 1,913 | 2,237 | 2,478 |  |  | 1,296 | 1,781 | 1,868 | 2,217 |  |
| SE | 8.4 | 4.6 | 6.5 | 6.0 | <.0001 |  | 6.0 | 4.6 | 6.9 | 8.4 | <.0001 |
| Total magnesium (mg)^2^ |  |  |  |  |  |  |  |  |  |  |  |
| Mean^3^ | 351.4 | 410.3 | 454.3 | 500.7 |  |  | 324.3 | 364.9 | 391.1 | 432.3 |  |
| SE | 1.2 | 0.6 | 0.9 | 0.8 | <.0001 |  | 0.8 | 0.6 | 0.9 | 1.1 | <.0001 |
| Vitamin D (mcg/day) |  |  |  |  |  |  |  |  |  |  |  |
| Mean^2^ | 4.0 | 9.1 | 13.8 | 19.3 |  |  | 6.6 | 9.0 | 10.9 | 13.8 |  |
| SE | 0.09 | 0.07 | 0.05 | 0.07 | <.0001 |  | 0.05 | 0.03 | 0.05 | 0.06 | <.0001 |

^1^ Least squares mean value, SE

^2^ Least squares mean value, SE, adjusting for total energy

**Supplementary Table 3. Sensitivity analyses: Association between calcium intake and distal colorectal cancer by screening arm allocation (intervention vs. control arm)**

| Calcium Intake (mg/day) | Distal colorectal cancer  in the intervention arm | |  | Distal colorectal cancer  in the control arm | |
| --- | --- | --- | --- | --- | --- |
|  | Cases | HR (95% CI) |  | Cases | HR (95% CI) |
| **All** | | | | | |
| <600 | 52 | 1.42 (0.97-2.07) |  | 89 | 1.43 (1.03-1.98) |
| 600-1200 | 135 | 1.00 (Ref) |  | 119 | 1.00 (Ref) |
| 1200-1600 | 51 | 0.74 (0.52-1.06) |  | 42 | 0.78 (0.54-1.13) |
| ≥1600 | 62 | 0.85 (0.58-1.25) |  | 30 | 0.71 (0.45-1.12) |
| *P* _trend_ |  | 0.06 |  |  | <0.01 |

^1^ Adjusted for age (continuous), sex, BMI (<25, 25-30, ≥30), education (less than high school, 12 years or completed high school, post high school training other than college, some college, college graduate, postgraduate), race (white, black, asian or others), family history of colorectal cancer (yes or no), cigarette (never smoked cigarettes, current or former), and total energy and vitamin D intake

^2^ Assigned the score j to the j^th^ level of calcium intake and evaluated the significance of Wald test

**Supplementary Table 4. Joint association between calcium intake category and magnesium category in relation to adenoma incidence (any, advanced/synchronous adenoma)**

| Ca Intake (mg/day) | Mg RDA^2^ | Any adenoma | |  | Advanced and/or synchronous adenoma | |
| --- | --- | --- | --- | --- | --- | --- |
|  | Threshold | Cases | OR (95% CI) |  | Cases | OR (95% CI) |
| <600 | < RDA | 127 | 0.94 (0.73-1.20) |  | 51 | 1.02 (0.69-1.50) |
| 600-1200 | < RDA | 272 | 1.00 (0.83-1.21) |  | 84 | 0.79 (0.57-1.08) |
| 1200-1600 | < RDA | 44 | 0.85 (0.60-1.20) |  | 16 | 0.82 (0.47-1.43) |
| ≥1600 | < RDA | 15 | 0.70 (0.41-1.19) |  | 7 | 0.89 (0.41-1.93) |
| <600 | ≥ RDA | 12 | 0.82 (0.44-1.53) |  | 4 | 0.79 (0.29-2.20) |
| 600-1200 | ≥ RDA | 273 | 1.00 (Ref) |  | 108 | 1.00 (Ref) |
| 1200-1600 | ≥ RDA | 175 | 0.81 (0.66-0.99) |  | 54 | 0.60 (0.43-0.86) |
| ≥1600 | ≥ RDA | 229 | 0.86 (0.69-1.06) |  | 76 | 0.70 (0.49-0.99) |

^1^ Adjusted for age (continuous), sex, BMI (<25, 25-30, ≥30), education (less than high school, 12 years or completed high school, post high school training other than college, some college, college graduate, postgraduate), race (white, black, asian or others), family history of colorectal cancer (yes or no), cigarette (never smoked cigarettes, current or former), hours spent in vigorous activities (less than1 hour/week, 1 hour/week, 2 hours/week, 3 hours/week, 4+ hours/week), and total energy and vitamin D intake
^2^ RDA: 320 and 420 mg/day for women and men, respectively
Calcium intake categories and Mg RDA categories (Below vs. Equal to or above RDA) were combined to create the following variables: Ca <600/Mg <RDA, Ca 600-1200/Mg <RDA, Ca 1200-1600/Mg <RDA, Ca >1600/Mg <RDA, Ca <600/Mg ≥RDA, Ca 600-1200/Mg ≥RDA (Reference category), Ca 1200-1600/Mg ≥RDA, Ca >1600/Mg ≥RDA. All other categories were compared to one reference category.

**Supplementary Table 5. Joint association between calcium intake category and magnesium category in relation to metachronous adenoma (any, advanced/synchronous adenoma)**

| Ca Intake (mg/day) | Mg RDA^2^ | Any adenoma | |  | Advanced and/or synchronous adenoma | |
| --- | --- | --- | --- | --- | --- | --- |
|  | Threshold | Cases | OR (95% CI) |  | Cases | OR (95% CI) |
| <600 | < RDA | 114 | 1.32 (0.89-1.95) |  | 58 | 1.46 (0.91-2.36) |
| 600-1200 | < RDA | 211 | 1.19 (0.87-1.62) |  | 112 | 1.28 (0.87-1.88) |
| 1200-1600 | < RDA | 35 | 1.25 (0.72-2.19) |  | 19 | 1.23 (0.63-2.40) |
| ≥1600 | < RDA | 12 | 0.95 (0.43-2.11) |  | 4 | 0.56 (0.18-1.79) |
| <600 | ≥ RDA | 10 | 1.91 (0.64-5.69) |  | 7 | 2.66 (0.79-8.90) |
| 600-1200 | ≥ RDA | 182 | 1.00 (Ref) |  | 86 | 1.00 (Ref) |
| 1200-1600 | ≥ RDA | 155 | 1.05 (0.76-1.46) |  | 77 | 1.08 (0.71-1.62) |
| ≥1600 | ≥ RDA | 136 | 0.86 (0.61-1.23) |  | 77 | 1.05 (0.68-1.63) |

^1^ Adjusted for age (continuous), sex, BMI (<25, 25-30, ≥30), education (less than high school, 12 years or completed high school, post high school training other than college, some college, college graduate, postgraduate), race (white, black, asian or others), family history of colorectal cancer (yes or no), cigarette (never smoked cigarettes, current or former), hours spent in vigorous activities (less than1 hour/week, 1 hour/week, 2 hours/week, 3 hours/week, 4+ hours/week), and total energy and vitamin D intake
^2^ RDA: 320 and 420 mg/day for women and men, respectively
Calcium intake categories and Mg RDA categories (Below vs. Equal to or above RDA) were combined to create the following variables: Ca <600/Mg <RDA, Ca 600-1200/Mg <RDA, Ca 1200-1600/Mg <RDA, Ca >1600/Mg <RDA, Ca <600/Mg ≥RDA, Ca 600-1200/Mg ≥RDA (Reference category), Ca 1200-1600/Mg ≥RDA, Ca >1600/Mg ≥RDA. All other categories were compared to one reference category.

**Supplementary Table 6. Joint association between calcium intake category and magnesium category in relation to incident colorectal cancer (all, proximal, and distal)**

| Calcium Intake | Mg RDA^2^ | All Colorectal cancer | |  | Proximal colon cancer | |  | Distal colorectal cancer | | | |
| --- | --- | --- | --- | --- | --- | --- | --- | --- | --- | --- | --- |
| (mg/day) | Threshold | Cases | HR (95% CI) |  | Cases | HR (95% CI) |  | Cases | | HR (95% CI) | |
| <600 | < RDA | 244 | 1.28 (1.03-1.58) |  | 120 | 1.09 (0.82-1.46) |  | 124 | | | 1.54 (1.13-2.09) |
| 600-1200 | < RDA | 296 | 1.18 (0.98-1.42) |  | 162 | 1.12 (0.87-1.44) |  | 134 | | | 1.26 (0.96-1.66) |
| 1200-1600 | < RDA | 53 | 0.87 (0.63-1.18) |  | 34 | 1.00 (0.68-1.48) |  | 19 | | | 0.68 (0.40-1.16) |
| ≥1600 | < RDA | 30 | 1.23 (0.82-1.84) |  | 17 | 1.35 (0.81-2.25) |  | 13 | | | 1.04 (0.52-2.06) |
| <600 | ≥ RDA | 30 | 1.27 (0.86-1.88) |  | 13 | 0.92 (0.51-1.68) |  | | 17 | | 1.74 (1.04-2.94) |
| 600-1200 | ≥ RDA | 261 | 1.00 (Ref) |  | 140 | 1.00 (Ref) |  | | 120 | | 1.00 (Ref) |
| 1200-1600 | ≥ RDA | 174 | 0.91 (0.74-1.11) |  | 100 | 0.95 (0.72-1.25) |  | | 74 | | 0.87 (0.64-1.17) |
| ≥1600 | ≥ RDA | 187 | 0.88 (0.71-1.09) |  | 104 | 0.93 (0.70-1.24) |  | | 79 | | 0.79 (0.57-1.09) |

^1^ Adjusted for arm, age (continuous), sex, BMI (<25, 25-30, ≥30), education (less than high school, 12 years or completed high school, post high school training other than college, some college, college graduate, postgraduate), race (white, black, asian or others), family history of colorectal cancer (yes or no), cigarette (never smoked cigarettes, current or former), hours spent in vigorous activities (less than1 hour/week, 1 hour/week, 2 hours/week, 3 hours/week, 4+ hours/week), and total energy and vitamin D intake
^2^ RDA: 320 and 420 mg/day for women and men, respectively
Calcium intake categories and Mg RDA categories (Below vs. Equal to or above RDA) were combined to create the following variables: Ca <600/Mg <RDA, Ca 600-1200/Mg <RDA, Ca 1200-1600/Mg <RDA, Ca >1600/Mg <RDA, Ca <600/Mg ≥RDA, Ca 600-1200/Mg ≥RDA (Reference category), Ca 1200-1600/Mg ≥RDA, Ca >1600/Mg ≥RDA. All other categories were compared to one reference category.
